# Genome Sequence of Acetobacter tropicalis DmPark25_167, a Bacterium Isolated from a Drosophila melanogaster Genetic Model of Parkinson’s Disease

**DOI:** 10.1128/MRA.01138-20

**Published:** 2021-01-07

**Authors:** Julie A. Goddard, Samara C. Petersen, Gerald B. Call, John M. Chaston

**Affiliations:** a Department of Plant and Wildlife Sciences, Brigham Young University, Provo, Utah, USA; b Department of Pharmacology, College of Graduate Studies, Midwestern University, Glendale, Arizona, USA; University of Arizona

## Abstract

Here, we report the genome of Acetobacter tropicalis DmPark25_167, a bacterial strain isolated from a Drosophila melanogaster *park^25^* mutant. The *park^25^* mutant is an established genetic model of Parkinson’s disease. DmPark25_167 has duplicated methionine metabolism and type IV secretion gene alleles compared with another strain of A. tropicalis.

## ANNOUNCEMENT

Acetobacter tropicalis is a species of acetic acid bacteria commonly associated with Drosophila melanogaster (1). Here, we report the genome sequence of A. tropicalis DmPark25_167, a strain cultured from homogenates of a D. melanogaster *park^25^* mutant, which is a model of Parkinson’s disease that was provided to the Call laboratory by Leo Pallanck (2). We also report genomic differences between DmPark25_167 and DmCS_006, another A. tropicalis strain. We present this genome in our continued efforts to understand the genomic basis for *Drosophila-*microbiota interactions.

The genome sequence of DmPark25_167 was obtained using the following approach. A pool of five whole-body *Drosophila park^25^* mutants (reared on the following diet formulation per 1 liter food: 0.957 liter water, 11.8 g yeast, 5.9 g soy flour, 5.9 g agar, 58.8 g yellow cornmeal, 35.3 ml light corn syrup, 35.3 ml molasses, 4.4 ml propionic acid, and 10 ml 10% methylparaben in 95% ethanol) were homogenized and plated onto modified MRS medium (3) in a Glendale, AZ, laboratory. Copper and tan colonies that grew were streaked for isolation, and their taxonomic assignment as *Acetobacter* species was confirmed by Sanger sequencing using primers based on the universal 27F and 1492R primers (27F, 5′-AGAGTTTGATCMTGGCTCAG-3′; 1492R, 5′-TACGGYTACTACCTTGTTACGACTT-3′). Following our previous methods (4), we prepared a genome sequencing library for an isolate with 100% 16S sequence identity to A. tropicalis DmCS_006, a strain of *Acetobacter* isolated from laboratory D. melanogaster Canton-S in Ithaca, NY (5). DNA was extracted using the Qiagen DNeasy Powerlyzer microbial kit, fragmented to 500 bp (NEBNext double-stranded DNA [dsDNA] Fragmentase [M0348L]), end repaired (NEBNext Ultra II end repair/dA-tailing module [E7546L]), ligated to Illumina sequencing adapters (NEBNext Ultra II ligation module [E7595L]), and size selected to ∼500 bp. Ligated target sequences were enriched with the KAPA library amplification kit (KK2621), size selected to 500 bp, and normalized to 5 ng/μl using Qubit measurements. The library was sequenced by 250-bp paired-end sequencing on a partial lane of an Illumina HiSeq 2500 instrument. The 1,828,193 paired-end reads that passed Illumina default quality filtering were assembled with Velvet version 1.2.10 (6). A k-mer length of 151 was chosen by testing k-mer lengths between 135 and 231 in increments of 8 and selecting an assembly that maximized the *N*_50_ value at an estimated nucleotide coverage of 114×. The final assembly contained 3,722,678 bp in 138 contigs and had an *N*_50_ value of 105,815 and a 55.5% GC content. The taxonomic assignment as A. tropicalis was verified using ANIm analysis in JSpeciesWS (7). DmPark25_167 contains ∼3,383 genes that code for 3,215 proteins, as annotated by the NCBI Prokaryotic Genome Annotation Pipeline (8). Default parameters were used for all software unless otherwise specified.

We compared the gene contents of DmPark25_167 and DmCS_006 using RAST 2.0 annotations (9). Relative to DmCS_006, DmPark25_167 has perfect and lower-similarity (<89%) versions of single-copy DmCS_006 methionine metabolism and type IV secretion genes. The type IV secretion genes are on a contig with a plasmid replication initiation protein, suggesting that they might be on a plasmid.

### Data availability.

This whole-genome shotgun project and raw reads have been deposited at DDBJ/ENA/GenBank under accession numbers JACAOJ000000000 and SAMN15399467, respectively.
